# Supplementary material for: Oncolytic Vaccinia Virus Augments T Cell Factor 1-Positive Stem-like CD8+ T Cells, Which Underlies the Efficacy of Anti-PD-1 Combination Immunotherapy
Source: Biomedicines. 2022 Mar 30;10(4):805. doi: 10.3390/biomedicines10040805 (PMC9027961; doi:10.3390/biomedicines10040805)
Supplement: Supplementary file 1 [file biomedicines-10-00805-s001.zip › biomedicines-1636885-supplementary.pdf]

Oncolytic vaccinia virus augments T cell factor 1-positive stem-like $CD8^+$ T cells, which underlies the efficacy of anti-PD-1 combination immunotherapy

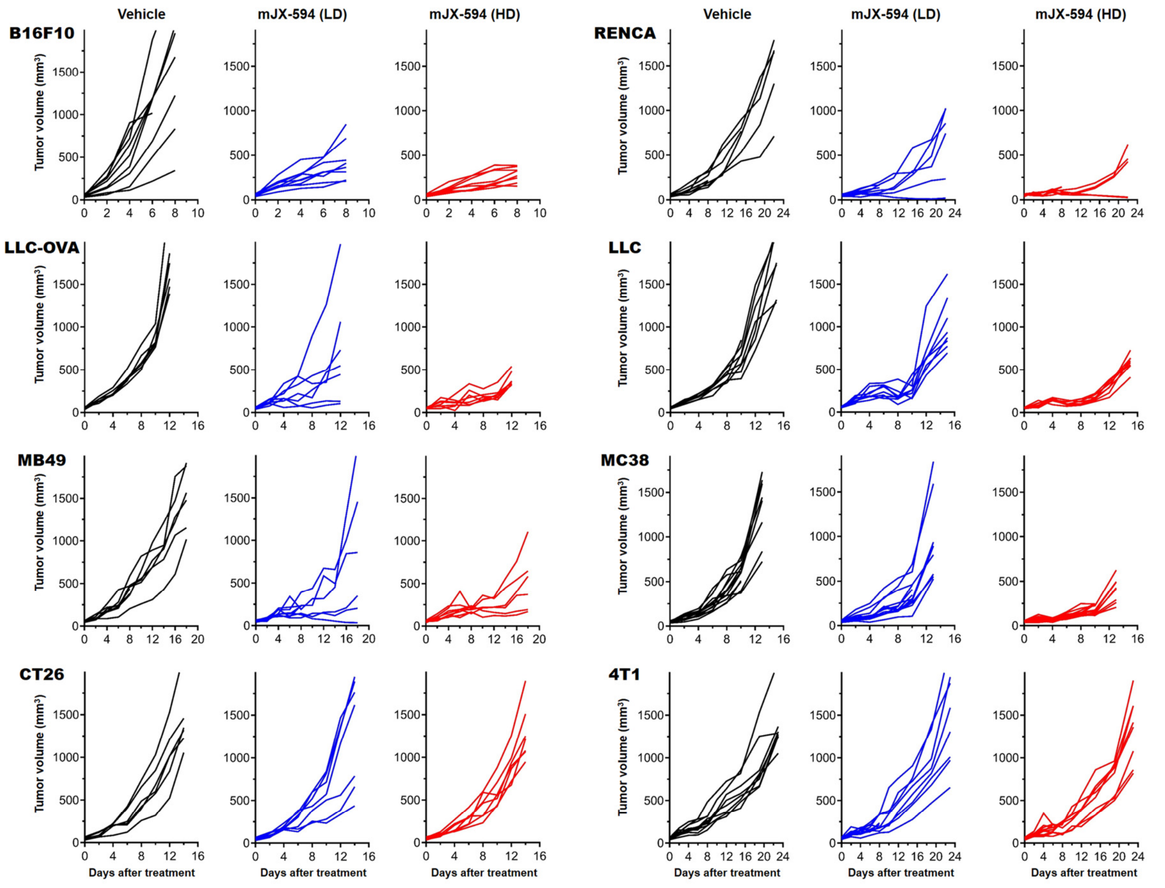

Supplementary Figure S1. Treatment efficacy of mJX-594 in syngeneic murine cancer models. Individual tumor growth curves of B16-F10, RENCA, LLC-OVA, LLC, MB49, MC38, CT26, and 4T1 tumors treated with vehicle, mJX-594 (LD), or mJX-594 (HD).

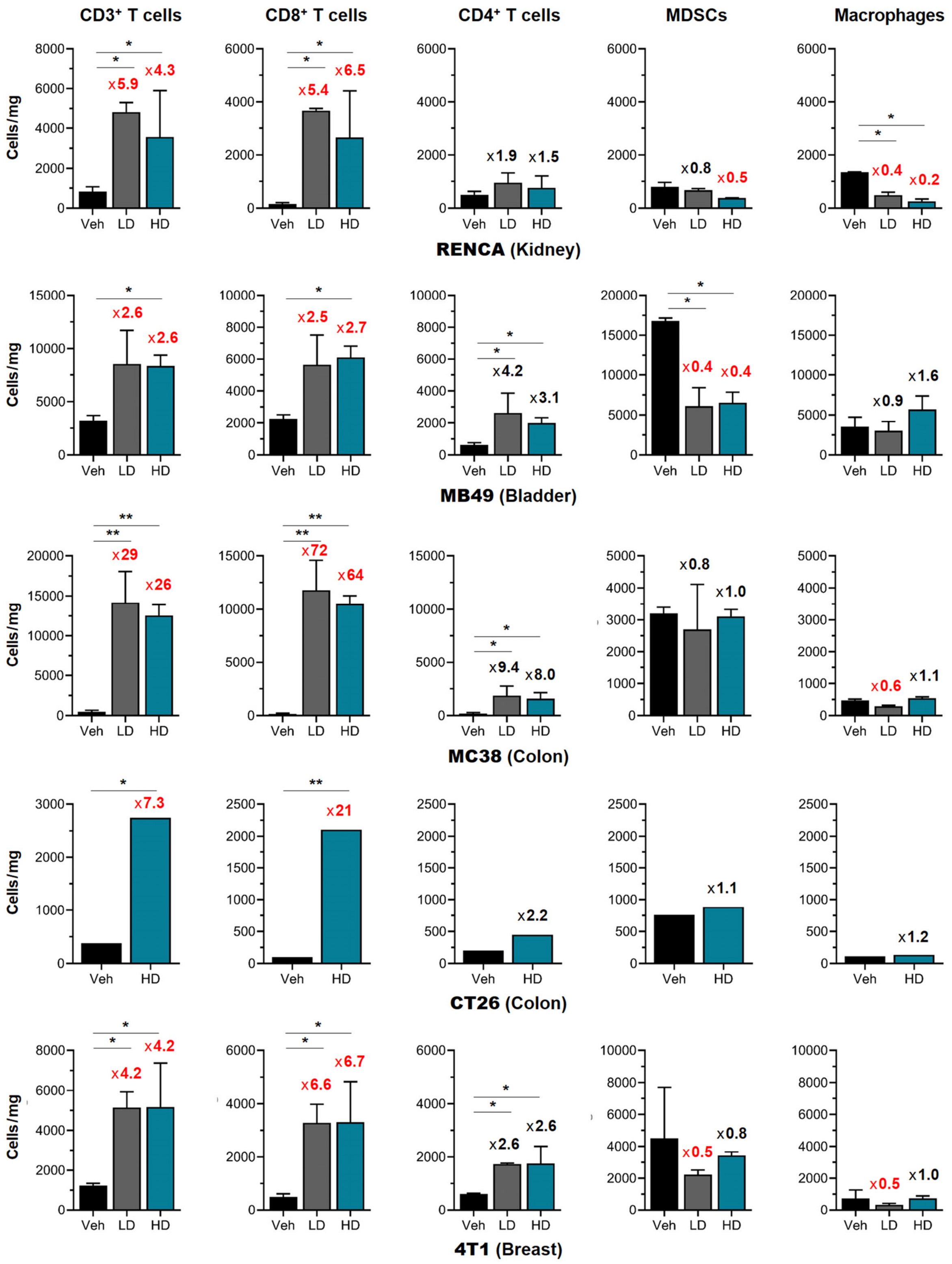

Supplementary Figure S2. Fold change of infiltrating leukocytes following mJX-594 treatment. The numbers of $CD3^+$ T cells, $CD8^+$ T cells, $CD4^+$ T cells, MDSCs, and macrophages per milligram of tumor mass among infiltrated leukocytes of RENCA, MB49, MC38, CT26, and 4T1 tumors on days 3 after the last mJX-594 treatment. Pooled data from

two experiments are shown (n = 6 per group). Values are mean±SD. *p<0.05, **p<0.005.

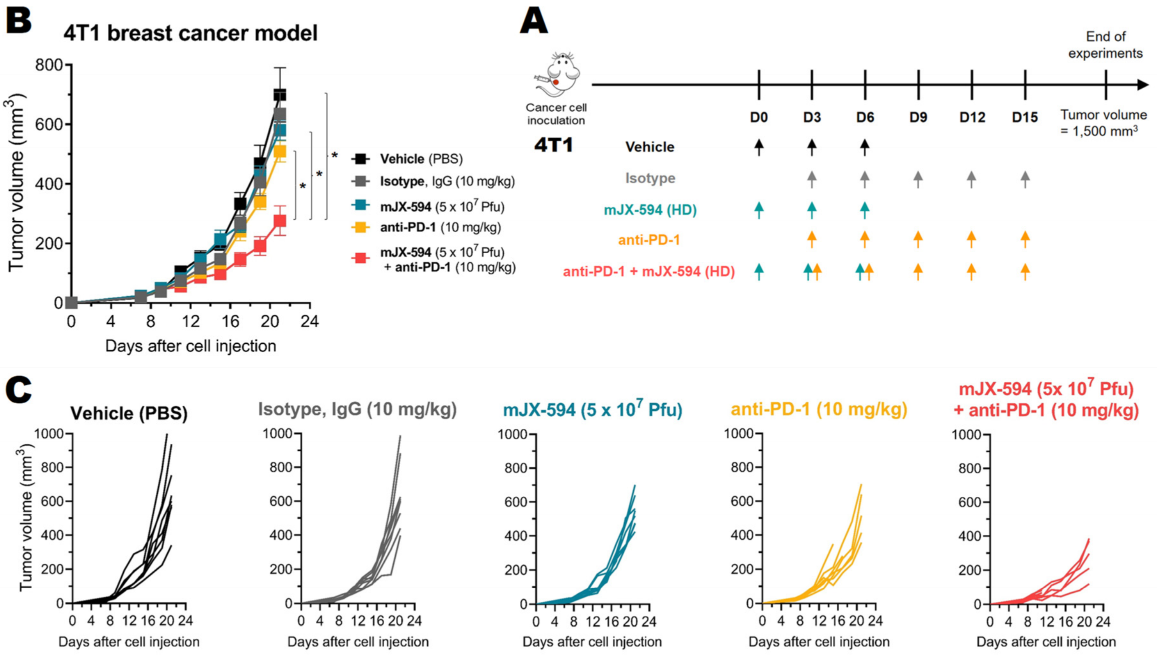

Supplementary Figure S3. Combinational treatment of anti-PD-1 with mJX-594 revealed anti-tumor effects in a 4T1 breast cancer model. (A) Combination treatment strategy. When 4T1 tumors reached 50–60 mm^3 in volume, mice were intratumorally administered 5 × 10^7 pfu of mJX-594 on days 0, 3, and 6. For combination therapy, anti-PD-1 antibody was intravenously injected five times at 3-day intervals, starting from the second treatment of mJX-594. (B) Average tumor growth curve of 4T1 tumor-bearing mice after mJX-594 and anti-PD-1 combination treatment (n = 7 per group). Group comparisons of tumor growth were carried out by two-way ANOVA with Bonferroni correction. Values are mean±SEM. *p<0.05. (C) Individual tumor growth curves of 4T1 tumor bearing mice after mJX-594 and anti-PD-1 combination treatment (n = 7 per group).

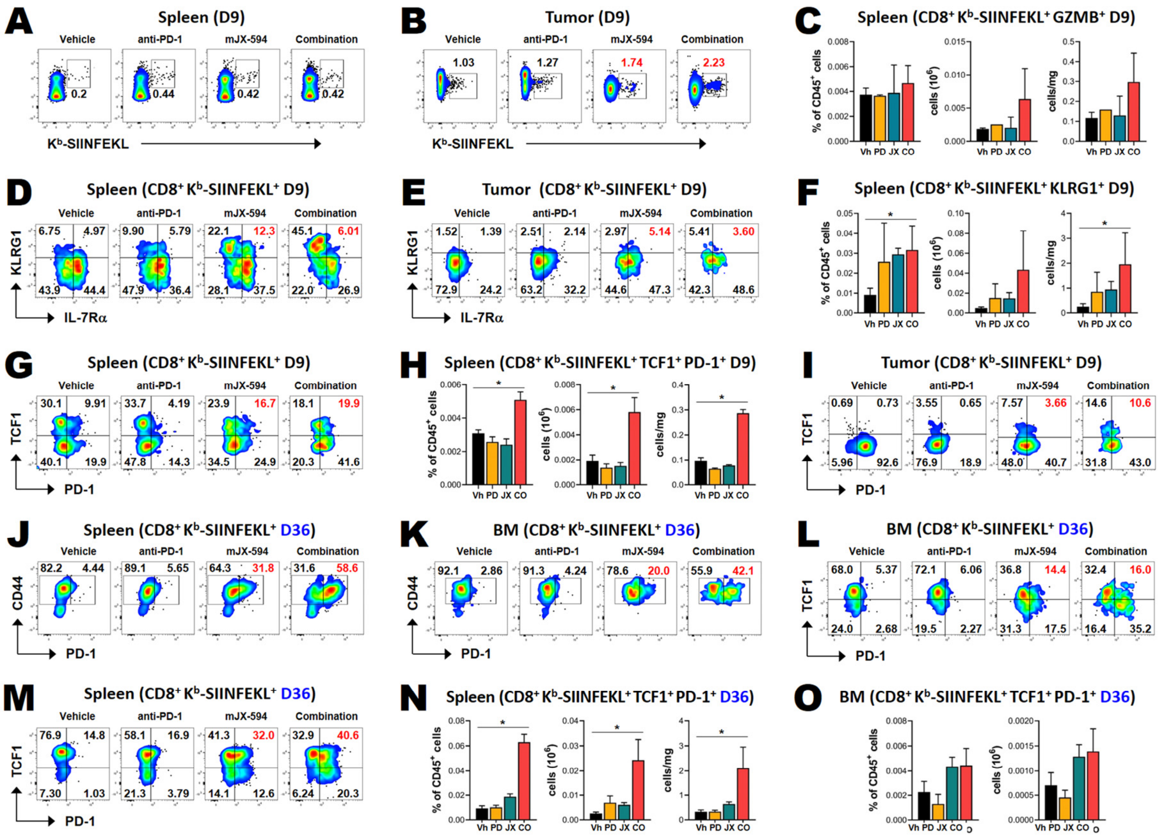

Supplementary Figure S4. Analysis of cancer neoantigen-specific CD8^{+} T cells following mJX-594, anti-PD-1, and combination therapy. When LLC-OVA tumors reached 50–60 mm^{3} in volume, the mice were intratumorally injected with vehicle or 5 × 10^{7} pfu of mJX-594 on days 0, 3, and 6. For combination therapy, anti-PD-1 antibody was intravenously injected five times at 3-day intervals, starting from the second treatment of mJX-594. OVA neoantigen-specific CD8^{+} (CD8^{+}K^{b}-SIINFEKL^{+}) T cells from the spleen, tumor, and bone marrow were analyzed by flow cytometry on days 9 and 36 after the first mJX-594 treatment. (A,B) Percentage of K^{b}-SIINFEKL^{+} cells among CD8^{+} T cells in spleen and tumor (D9). (C) Relative proportion among infiltrating leukocytes, total cell numbers, and number of cells per unit gram of tissue of K^{b}-SIINFEKL^{+} GzmB^{+} effector CD8^{+} T cells in spleen (D9). (D,E) Memory precursor effector cells (IL-7Rα^{+}KLRG1^{int}) and short-lived effector cells (IL-7Rα^{-}KLRG1^{high}) among CD8^{+} T cells in spleen and tumor (D9). (F) K^{b}-SIINFEKL^{+} KLRG1^{+} effector CD8^{+} T cells in spleen (D9). (G,H,I) K^{b}-SIINFEKL^{+} TCF1^{+}PD-1^{+} stem-like CD8^{+} T cells in spleen and tumor (D9). (J,K) K^{b}-SIINFEKL^{+} CD44^{+}PD-1^{+} memory-phenotype CD8^{+} T cells in spleen and bone marrow (D36). (L,M,N,O) K^{b}-SIINFEKL^{+} TCF1^{+}PD-1^{+} stem-like CD8^{+} T cells in spleen and tumor (D36). Pooled data from two experiments are shown (n = 6 per group). *$p<0.05$. CO, combination therapy; GZMB, granzyme B; JX, mJX-594; OVA,

ovalbumin; PD, anti-PD-1; Vh, vehicle.
